# Visual Place Recognition Based on Dynamic Difference and Dual-Path Feature Enhancement

**DOI:** 10.3390/s25133947

**Published:** 2025-06-25

**Authors:** Guogang Wang, Yizhen Lv, Lijie Zhao, Yunpeng Liu

**Affiliations:** 1 College of Information Engineering, Shenyang University of Chemical Technology, Shenyang 110142, China; yz_lv000@163.com (Y.L.); zlj_lunlun@163.com (L.Z.); 2 Shenyang Institute of Automation, Chinese Academy of Sciences, Shenyang 110016, China; ypliu@sia.cn

**Keywords:** visual place recognition, DINOv2 model, two-way feature enhancement, adaptive weighting

## Abstract

Aiming at the problem of appearance drift and susceptibility to noise interference in visual place recognition (VPR), we propose DD–DPFE: a Dynamic Difference and Dual-Path Feature Enhancement method. Embedding differential attention mechanisms in the DINOv2 model to mitigate the effects of process interference and adding serial-parallel adapters allows efficient model parameter migration and task adaptation. Our method constructs a two-way feature enhancement module with global–local branching synergy. The global branch employs a dynamic fusion mechanism with a multi-layer Transformer encoder to strengthen the structured spatial representation to cope with appearance changes, while the local branch suppresses the over-response of redundant noise through an adaptive weighting mechanism and fuses the contextual information from the multi-scale feature aggregation module to enhance the robustness of the scene. The experimental results show that the model architecture proposed in this paper is an obvious improvement in different environmental tests. This is most obvious in the simulation test of a night scene, verifying that the proposed method can effectively enhance the discriminative power of the system and its anti-jamming ability in complex scenes.

## 1. Introduction

Visual Place Recognition (VPR) is an important research direction in the field of computer vision and robotics, aiming to determine the location of a device by matching images with a series of databases. VPR is commonly used in the fields of robot navigation, autonomous driving, and augmented reality [1]. There are a number of serious challenges in visual location recognition tasks, including changing lighting conditions [2], seasonal changes and time lapse [3], weather fluctuations [4], perspective shifts [5], and dynamic object occlusion [6], which can significantly interfere with the accuracy and robustness of image matching.

Current research approaches in visual location recognition follow two main technical routes: the classification paradigm and the retrieval paradigm. Under the classification paradigm, researchers achieve recognition by constructing location classification models. CosPlace [7] constructs classifier training descriptors through geographic region division. D-Cosplace [8] introduces distributed training to improve model generalization. Hussaini et al. [9] draws on the classification idea to organize data and models, but the core is still retrieval. The classification paradigm has inherent flaws: there is a semantic divide between the continuity of geographic scenes and the discrete nature of classification labels, leading to insufficient zero-sample generalization for untrained regions. Mainstream research methods more often regard VPR as a retrieval problem, and their technical routes focus on constructing feature spaces with strong discriminative properties to support similarity matching. Li et al. [10] proposed the PPT-Hashing framework, which employs hash coding to achieve efficient image retrieval. Wu et al. [11] combined SCA and GIA to propose GICNet, which enhances the model’s feature extraction and feature aggregation capabilities to further improve the retrieval performance.

With the increase of task complexity and the deepening of practical application requirements, regardless of the adoption of classification or retrieval technology routes, in recent years the mainstream methods have widely adopted deep learning models as the basic support. Traditional feature matching methods such as SIFT [12] and SURF [13] are gradually being replaced by new methods based on deep learning. With the rapid development of the Transformer architecture in the field of computer vision, by virtue of its advantages in spatial feature modeling and global dependency capturing, it shows excellent performance in tasks such as image matching, which is especially suitable for scenarios that are highly dependent on contextual understanding such as VPR. In this context, academics have conducted systematic research on structural adaptation and feature enhancement of Transformer in VPR tasks, which has contributed to the continuous progress in this field. Zhu et al. [14] were the first to verify the performance advantage of the underlying Transformer backbone network in VPR tasks. Its global modeling capability significantly outperforms that of the traditional CNN backbone network. Keetha et al. [15] proposed DINO/DINOv2-driven ViT as the base backbone network on this basis, establishing a new performance benchmark for the VPR task. To further enhance the characterization capability of the backbone network, Lu et al. [16] achieved efficient migration of pre-trained models and task adaptation by adding adapters inside the Transformer encoder. The team further proposed multi-scale convolutional adapters in the concurrent work [17], which significantly improved the performance of the VPR task by fusing the local a priori and global modeling capabilities in the backbone network performance. Zhang et al. [18] proposed introducing the RGA attention mechanism into the feature fusion and enhancement module to model the fused features as a whole so as to effectively suppress redundant information and noise interference. Liu et al. [19] introduced POD attention between the feature extraction module and the feature aggregation module to suppress the high-frequency noise present in the shallow network by means of attention focusing. Although such methods enhance the robustness of feature representation to a certain extent, most of their suppression of noise occurs after the features have already been generated, failing to intervene directly in the process of feature formation, resulting in noise that may have propagated at an early stage and affected the quality of subsequent representations. Notably, studies have shown the potential limitations of the attention mechanism that comes with Transformer. Ye et al. [20] found through visual analysis that traditional Transformer often suffers from distraction in visual tasks, i.e., part of the attention head is focused on contextual information that is not relevant to localization. To solve this problem, they innovatively proposed a differential attention mechanism that effectively suppresses attentional noise and strengthens the feature response in key regions by establishing an adaptive correction module for the distribution of attention. Although this mechanism shows significant advantages in language models, its generalization efficacy in the task of cross-view visual place recognition is still to be studied in depth.

In the research direction of global and local feature representation optimization, scholars generally focus on improving the discrimination and robustness of feature representation to further enhance the matching accuracy in visual localization tasks. For the construction of global features, most of the existing mainstream methods achieve feature map dimension reduction through feature aggregation techniques, specifically using pooling operations such as GeM [21], mean pooling [22], NetVLAD [23], etc., to encode higher-order statistics of features to obtain global feature representations. Wang et al. [24] achieved adaptive attentional weighting by cross-layer aggregation of multi-scale patch tokens from the Transformer encoder, which is the most effective way to optimize feature representation. Adaptive attention assigns weights to patch tokens, then realizes the global feature representation of context-awareness. Lu et al. [17] first divided the patch tokens’ output from the backbone network into different scales and performed pooling and cross-scale feature splicing, then realized cross-region feature interactions through the self-attention mechanism of the Transformer encoder. The structure was able to effectively realize the high-level integration of global context information. In terms of local feature modeling, research focuses more on improving the responsiveness and robustness of local features to critical regions. Garg et al. [25] revealed that feature matching in the VPR task is sensitive to the noise in non-overlapping regions and proposed a method to enhance the focusing ability on critical regions through a feature weighting mechanism. Kannan et al. [26] proposed a multi-scale patch fusion method to enhance the focusing ability on critical regions by integrating image features at different scales to improve the matching performance of images under scale transformation and viewpoint change. Khaliq et al. [27] revealed the systematic risk of feature redundancy in the VPR task: the proliferation of repetitive patterns in the feature space will lead to discriminative degradation and mismatch propagation. It is proposed to construct an adaptive suppression weight matrix in the feature similarity space through a dynamic feature competitive learning mechanism, dynamically adjust the contribution of local features to the aggregated vector, suppress the degradation of the representations triggered by the high-frequency repetitive patterns, and make the aggregated vector closer to low-conflict and high-discriminative local features.

The main contributions of the work in this paper are as follows:

(1) We innovatively construct a dynamic differential DINOv2 model, which introduces the differential attention mechanism to the visual place recognition task for the first time. The mechanism effectively decouples the noise from the key features, adaptively adjusts the noise suppression strength through dynamic parameters, and significantly enhances the model’s adaptability to environmental changes and cross-domain generalization ability.
(2) We propose a dual-path feature enhancement module. The global path adopts a dynamic hierarchical fusion mechanism, retains the initial feature statistics through multilevel semantic association modeling, effectively solves the problem of apparent offset caused by drastic lighting/viewing angle changes, and significantly improves the global consistency of cross-scene representations. Local paths are weighted by adaptive aggregation to suppress repetitive texture interference and enhance discriminative local detail representation. The dual-path cooperative mechanism optimizes global–local features and significantly improves cross-domain matching performance.
(3) Comprehensive experiments on DD–DPFE on six mainstream VPR datasets including extreme weather/light conditions show that DD–DPFE exhibits advantages on multiple datasets, especially in challenging scenes.

The other sections are structured as follows: Section 2 describes in detail the research methodology proposed; Section 3 is the experimental part, which verifies the superiority of this paper’s methodology through comparative experiments and further analyzes the contribution of each module to the overall performance in conjunction with ablation experiments; and in Section 4 the paper is summarized.

## 2. Methodology

**Figure 1:**
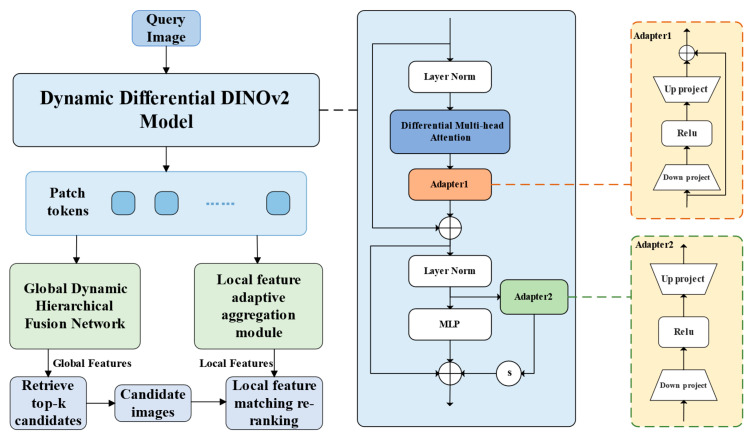
DD–DPFE Structure.

We propose a DD–DPFE network. Its structure is detailed in Figure 1. In the dynamic differential DINOv2 model, the feature aggregation process of multi-head attention is reconstructed by a differential attention mechanism to reduce the interference of noise on high-dimensional feature expression, and the dynamic adapter module is embedded to realize efficient parameter migration and task adaptation. A dual-path feature enhancement module including a global dynamic hierarchical fusion module and a local feature adaptive weighted aggregation module is proposed. The global dynamic hierarchical fusion module constructs hierarchical feature dynamic fusion on the basis of initial global features generated by GeM pooling so that the model can enhance the cross-domain semantic association modeling capability while maintaining the integrity of initial statistical features. The local feature adaptive weighted aggregation module improves the model’s accuracy in capturing and processing local details by reducing the negative impact of duplicate regions on feature matching.

### 2.1. Dynamic Differential DINOv2 Model

DINOv2 employs Vision Transformer (ViT) as the backbone network to achieve feature consistency modeling across image views through a self-supervised joint embedding learning framework. In order to improve the model’s adaptability and parameter migration efficiency in downstream tasks and to improve the susceptibility of the standard multi-head attention to noise interference, we propose a dynamic differential DINOv2 model, the structure of which is shown in Figure 1. Its core feature extraction process is described as follows.

Given an input image, the backbone network first performs a transformation by embedding, dividing the input image 224×224 into patches of size 14×14, yielding 16×16 patches. Each patch is then mapped by linear transformation to obtain a vector of length 1024. The final sequence of tokens (patch tokens and additional learnable global tokens and class tokens) is embedded with positional information and then fed into the optimized Transformer encoder for processing to generate feature representations. Assuming the input is $z_{l-1}$, the encoding process for the lth Transformer module is as follows:

(1) $$z_{l}^{\prime }=Adapter1(MHA^{diff}(LN(z_{l-1})))+z_{l-1}$$

(2) $$z_{l}=MLP(LN(z_{l}^{\prime }))+s\cdot Adapter2(LN(z_{l}^{\prime }))+z_{l}^{\prime }$$

Here, $z_{l}$ denotes the output of the lth encoder block, ${z_{l}}^{\prime }$ denotes the output before layer normalization, s is the scaling factor, Adapter denotes the adapter, ${MHA}^{diff}(\cdot )$ denotes the differential multi-head attention, and LN(⋅) denotes layer normalization.

Each Transformer encoder has two adapters: Adapter1 is a serial adapter after the differential attention layer with internal hopping connections. Adapter2 is a parallel adapter connected in parallel to the MLP layer.

**Figure 2:**
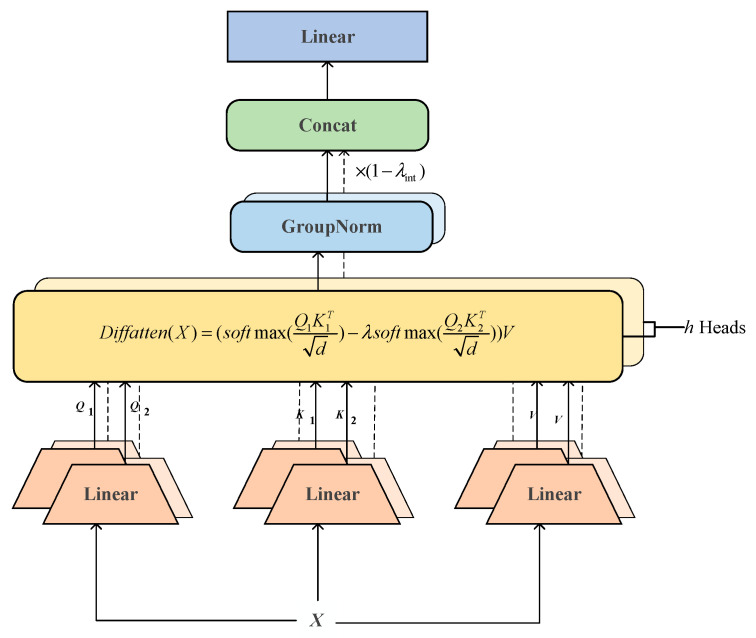
Differential Attention.

The differential attention mechanism is shown in Figure 2. It centers on constructing the difference computation of two kinds of attention weights. $A_{1}$ and $A_{2}$ are the scores of the attention of two different query and key pairs ($Q_{1},K_{1}$) and ($Q_{2},K_{2}$), which come from different parts of the input X. Through the difference computation, it tries to distinguish the effective attention scores in $A_{1}$ from the redundant or unnecessary attention scores of $A_{2}$ to differentiate them, thus effectively eliminating redundant information as well as common-mode noise in the input and enhancing the effective attention weight distribution.

It uses Group Norm to emphasize that the layer normalization $LN\left(\cdot \right)$ is enacted independently on top of each head to ensure that the feature distribution of each head is independent and stable, which can eliminate the difference in output variance between different heads, thus reducing the noise generated by inconsistent computations performed by different heads. The calculation process is as follows:

(3) $$A_{1}=\frac{Q_{1}K_{1}^{T}}{\sqrt{d}}$$

(4) $$A_{2}=\frac{Q_{2}K_{2}^{T}}{\sqrt{d}}$$

(5) $$DiffAtten\left(X\right)=\left(softmax\left(A_{1}\right)-\lambda \cdot softmax\left(A_{2}\right)\right)V$$

(6) $$\lambda =exp\left(\lambda_{q1}\cdot \lambda_{k1}\right)-exp\left(\lambda_{q2}\cdot \lambda_{k2}\right)+\lambda_{int}$$

(7) $$Head_{i}^{diff}=DiffAtten\left(X\right)$$

(8) $$\bar{Head_{i}^{diff}}=\left(1-\lambda_{int}\right)\cdot LN\left(Head_{i}^{diff}\right)$$

(9) $$MHA{\left(X\right)}^{diff}=Concat\left(\bar{Head_{1}^{diff}},\bar{Head_{2}^{diff}},\ldots ,\bar{Head_{h}^{diff}}\right)W_{o}$$

where the query-key dot product directly governs the value of λ, which serves as a dynamic adjustment factor, $\lambda_{q1}$, $\lambda_{q2}$, $\lambda_{k1}$, $\lambda_{k2}$ are the learnable vectors, and $\lambda_{int}$ stands for constants located between 0 and 1 used to initialize the λ.

### 2.2. Dual-Path Feature Enhancement Module

#### 2.2.1. Global Dynamic Hierarchy Fusion Module

In order to prevent the loss of deep spatial correlation information, we propose a global dynamic hierarchical fusion module that implements secondary feature refinement through dynamic fusion of the Transformer encoder on the basis of GeM pooling to generate the initial global feature representation and systematically optimizes the long-range dependencies in the feature space. The framework strengthens the semantic association between features through the self-attention mechanism, effectively eliminates redundant information interference, and ultimately generates light, robust features with strong discriminative properties to enhance the stability and matching accuracy of cross-view image retrieval.

**Figure 3:**
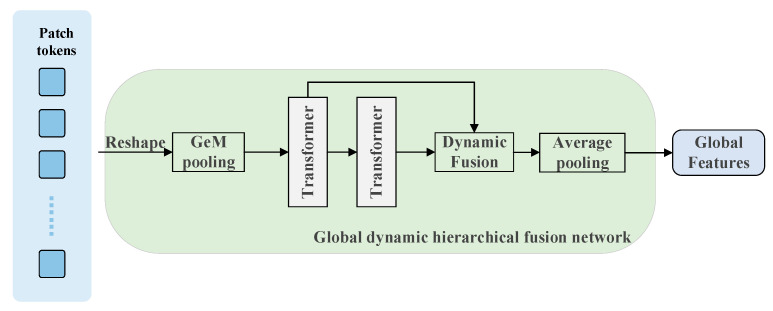
Global Dynamic Hierarchical Fusion Network.

The structure of the global dynamic hierarchical fusion network is constructed as shown in Figure 3, which contains three stages.

(1) Feature normalization and flexible aggregation

The ViT output patch feature matrix is $X\epsilon R^{B\times P\times D}$, where B is the number of batches, P is the number of patches, and D is the feature dimension. The L2 normalization is first performed for each patch and the public presentation is shown in (10).

(10) $$X_{norm}=\frac{X}{{\left\|X\right\|}_{2}}$$

The initial global descriptor is then constructed by GeM pooling as shown in (11), where i denotes the *i*th patch and the value of *P* controls the flexibility of feature aggregation: when P=1, GeM degrades to average pooling; when P→∞, GeM approximates maximal pooling and achieves successive transitions from fine-grained to salient features.

(11) $$X_{GeM}={\left(\frac{1}{P}\sum_{i=1}^{P}{\left(X_{norm,i}\right)}^{P}\right)}^{\frac{1}{P}}$$

(2) Dynamic fusion

The preliminary global features $X_{GeM}\epsilon R^{B\times P\times D}$ lack spatial semantics for dynamic interactions, although they possess global statistical properties. For this reason, the Transformer encoder is introduced to further optimize and construct a dynamic interaction network via the formulas shown in (12) and (13), where L is the number of encoder layers, $H^{\left(l\right)}$ denotes the output of the *l*th encoder block, and $w\in R^{L}$ is the learnable weight parameter used to achieve the dynamic balance of hierarchical features.

(12) $$H^{\left(l\right)}=Transformer\left(H^{\left(l-1\right)}\right),l\leq L$$

(13) $$H_{fused}=\sum a_{l}H^{\left(l\right)},a=softmax\left(w\right)$$

(3) Feature refinement and compression

The average pooling and normalization operation is performed for the optimized global features, and the formula is shown in (14), where $H_{fused}^{\left(p\right)}$ denotes the feature extracted from the pth patch. This operation eliminates feature redundancy through spatial dimension compression, enhances the representation’s tightness while preserving discriminative semantic information, and finally outputs a compact description with illumination invariance and viewpoint robustness.

(14) $$g=L2Norm\left(\frac{1}{P}\sum_{p=1}^{P}H_{fused}^{\left(p\right)}\right)$$

#### 2.2.2. Local Adaptive Weighted Aggregation Module

**Figure 4:**
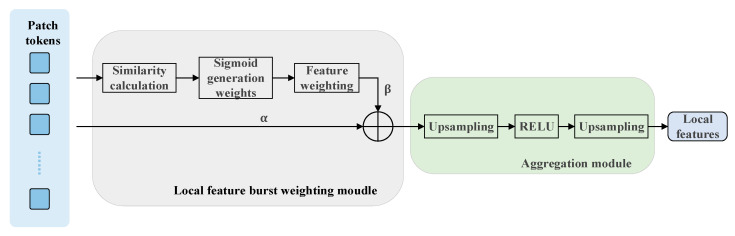
Local Feature Adaptive Weighted Aggregation Module.

High-frequency local patterns (e.g., repeating textures) are prone to triggering the sudden response of features, and there is a risk of causing model overfitting, which needs to be targeted. As shown in Figure 4, to address the interference brought by local repetitive features to the VPR task, we propose an adaptive weighted aggregation module for local features, which first suppresses the over-response of repetitive regions through the bursty weighting mechanism, then utilizes the feature aggregation module to fuse the multi-scale contextual information to generate denser local features with stronger discriminative properties so as to improve the matching accuracy in the reordering stage.

First, a matrix T consisting of N patch tokens is used to generate a similarity matrix S by calculating the similarity of all tokens via Equation (15). Next, the learnable parameters slope and offset are introduced to dynamically adjust the similarity distribution via Equation (16). Subsequently, the sigmoid function σ(⋅) in Equation (17) is utilized to map the adjusted similarity values between [0, 1] to generate the weights W. The weight matrix is then weighted with the original tokens as shown in Equation (18) to obtain the weighted tokens. Finally, the weighted tokens are weighted as shown in Equation (19). With the original tokens as shown in Equation (19), the weighted tokens are fused with the original tokens to generate the enhanced feature sequence F. This staged fusion strategy not only preserves the discriminative semantics of the original features but also effectively attenuates the noise interference caused by the repetitive regions.

(15) $$S=TT^{T}$$

(16) $$S_{adjusted}=slope\times S+offset$$

(17) $$W=\sigma \left(S_{adjusted}\right)$$

(18) $$tokens_{wight}=T\cdot W$$

(19) $$F=\alpha \cdot tokens+\beta \cdot tokens_{wight}$$

The dimensionality of the output F of the bursty weighting module is 16×16×1024. Here exists the problem of feature sparsity, and for a VPR task that requires high-resolution local features, we gradually compress the dimensionality of the feature channel from 1024 to 128 dimensions through the feature aggregation module (two up-convolutional and RELU activations) to produce dense local features to improve the performance of using local reordering of the marquee positions.

### 2.3. Search Method

In this paper, a two-stage hybrid retrieval framework is used, as shown in Figure 1. The first stage measures the similarity by calculating the L2 distance between the global feature vector q of the query image and the feature vector $d_{i}$ of the image in the database, as shown in (20). This stage is based on the FlatL2 implementation provided by Faiss, which quickly filters Top-k candidate images in a large-scale database by violent search to complete the preliminary coarse retrieval.

(20) $$Distance\left(q,d_{i}\right)={\left\|q-d_{i}\right\|}_{2}^{2}$$

To overcome ranking inaccuracies, the secondary processing phase employs local feature correlation analysis for candidate set optimization. Spatial alignment is performed by computing cosine similarities between the query descriptors $M_{q}\epsilon R^{B\times P\times D}$ and candidate descriptors $M_{c_{k}}\epsilon R^{B\times P\times D}$ at each position, following the metric defined in Equation (21). W×H denotes the spatial resolution, C denotes the number of feature channels, and (i,j) denotes the position index, and k denotes the index of the candidate image.

(21) $$s_{\left(i,j\right)}^{\left(k\right)}=\frac{M_{q}^{\left(i,j\right)}\cdot M_{c_{k}}^{\left(i,j\right)}}{\left\|M_{q}^{\left(i,j\right)}\right\|\left\|M_{c_{k}}^{\left(i,j\right)}\right\|}$$

Finally, the similarity of each candidate image at all positions is aggregated and averaged as the final score of the image. The candidate images are sorted in descending order according to the final score, and the optimized retrieval results are the output. The calculation formula is shown in Equation (22).

(22) $$score_{k}=\frac{1}{WH}\sum_{i=1}^{W}\sum_{j=1}^{H}s_{\left(i,j\right)}^{\left(k\right)}$$

## 3. Experiments

### 3.1. Dataset and Evaluation Indicators

In order to comprehensively evaluate the performance of this paper’s method in complex real-world scenarios, six representative and challenging VPR public datasets were selected as the evaluation benchmarks, which mainly include Pitts30k, MSLS, Nordland, AmsterTime, SF_XL, and SVOX. These datasets cover a wide range of real-world environment variation factors, such as illumination, viewing angle, seasonal changes, weather changes, and dynamic object interference, which can fully verify the robustness and generalization ability of the model in different dimensions. The specific information is shown in Table 1.

We adopted the recall rate (R@N) as a metric for evaluating the performance of VPR, where R@N refers to the probability that a correctly matched target location appears among the first N retrieval results in the query image. Specifically, R@1 indicates whether the first ranked retrieval result is the target location, and R@5 indicates whether the target location appears in the first five retrieval results. By calculating the metrics such as R@1 and R@5, the performance of the algorithm can be comprehensively evaluated under different difficulties and scenarios.

### 3.2. Implementation Details

We used DINOv2’s ViT-L/14 (1024 dimensions) as the base model. Experiments were performed on an NVIDIA GeForce RTX 4070 using Pytorch 2.0.0. All images were resized to 224×224 for training and evaluation, and the reordering was performed among the top 100 candidate images with the interval m=0.1 set. The bottleneck ratio of the adapter in the ViT block was 0.5, the scaling factor s=0.2, $\lambda_{int}=0.5$ in the differential attention, and the local feature aggregation module used a convolution of 3×3, where stride=2 and padding=1. The Adam optimizer training process (lr=0.00001, batch = 4) terminates automatically upon detecting no validation R@5 improvement over three successive epochs. The model in this paper was trained on the MSLS dataset, and the weights of the model were fine-tuned at Pitts30k.

### 3.3. Comparison with State-of-the-Art Methods

We compared the method in this paper with several other state-of-the-art VPR methods, including three one-stage methods using global feature retrieval: CosPlace [7], EigenPlace [28], and CircaVPR [17], alongside two-stage reordering-enhanced architectures including R^2^Former [14] and SelaVPR [16]. Among them, CircaVPR, R^2^Former, and SelaVPR are Transformer-based methods and CosPlace and EigenPlace are CNN-based methods, all of which have Resnet and VGG in their backbone networks. CosPlace’s backbone training network also contains a CCT and Transformer network. In this paper, the backbone network with the best effect in the literature was selected for the comparison test.

Table 2 systematically summarizes the architecture and training dataset details of the network models used for the VPR comparison experiments in this study. The test results for the baseline assessment data are presented in Table 3 and Table 4. These results present both optimal values (bolded) and suboptimal values (underlined), where “ave” indicates the average value, with all numerical results retained to one decimal place. It should be noted that all comparison experiments were conducted using unified hardware equipment for training and testing, which has led to slight deviations in some results compared to the original literature-reported data.

As demonstrated by the quantitative assessments in Table 3 and Table 4, DD–DPFE shows significant advantages in cross-scene generalization ability and extreme environment robustness, which are analyzed as follows:

In the benchmark dataset comparison, the proposed method achieved an overall lead on the Pitts30k dataset with 93.3% R@1 and 96.9% R@5, verifying its high-precision positioning capability in large-scale urban scenarios. At the same time, in extreme environment scenarios, its adaptability to long time spans (AmsterTime dataset R@1 is 59.3%) and its ability to express features of complex urban scenes (SF_XL (v1) dataset R@1 is 81.1%, surpassing most comparison methods) were more prominent. Although it was slightly less than the optimal method on the Nordland and MSLS-test datasets, its overall performance was stable, reflecting a good balance.

In the test of multiple weather scenes, the technical advantages of this method were further highlighted. In night scenes, the R@1 of 90% far exceeded that of CircaVPR (80.0%) and SelaVPR (71.6%). In rainy and snowy weather, the R@1 of 94.8% and 96.8%, respectively, set new performance records, which were 3.9% and 3.4% higher than the suboptimal methods. In strong light conditions, the R@1 of 92.9% far exceeded other advanced methods, indicating its strong robustness to low light, strong light, occlusion, and noise interference. At the same time, under overcast conditions, R@1 reached 96.3%, and the average R@1 of all weather scenes was as high as 94.2%, which is 5–25.7% higher than the comparison method. The R@5 index generally exceeded 92.9%, which verifies the effective trade-off between the precision and recall stability of the method.

Through the above experimental comparison and analysis, it can be seen that the proposed method has significant advantages in cross-dataset generalization, extreme weather robustness, and complex scene adaptability. It not only breaks through the bottleneck of large-scale positioning with the highest accuracy in urban scenes such as Pitts30k and SF_XL (v1) but also refreshes the performance record with more than 90% R@1 under extreme conditions such as night, rain, and snow. At the same time, it maintains a comprehensive lead in changeable weather such as cloudy days and strong light, providing an effective solution for visual positioning in actual complex environments.

### 3.4. Ablation Study

#### 3.4.1. Effect of Core Module Ablation

To scientifically evaluate the effectiveness of the three key enhancement modules proposed in this paper, systematic ablation experiments were designed under several typical cross-domain and complex weather scenarios. Specifically, based on the unified feature aggregation backbone structure, the dynamic differential attention mechanism (A), the local feature adaptive weighting module (W), and the global dynamic hierarchical fusion strategy (T) were gradually introduced, and several combination models were constructed to evaluate their performances one by one. The experimental results are shown in Table 5 and Table 6.

Based on the above ablation experiment results conducted on different datasets, the effectiveness and synergy of the global feature optimization module (T), the local burst weighting module (W), and the backbone network attention mechanism (A) are verified. The experimental results are analyzed as follows.

First, the introduction of the differential attention mechanism significantly reduces the attention noise on multiple VPR datasets and shows excellent performance. However, when dealing with the challenges of the AmsterTime dataset (involving long-term scene changes) and the SF_XL dataset (including dynamic viewpoint interference), as well as when dealing with some extreme weather scenes in the SVOX dataset (such as night, rain, and snow), the performance of the mechanism declined, which reveals the shortcomings of the model in terms of fine-grained matching accuracy, dynamic object robustness, cross-viewpoint consistency, and long-term environmental adaptability.

Subsequently, a local burst weighting module was introduced, which alleviated the above problems to a certain extent and strengthened the model’s local feature processing capabilities. The performance on the night scene was significantly improved to 76.7 (+2.5%), and the performance of all weather scenes tended to be stable, verifying its ability to suppress local feature redundancy. At the same time, the performance on the AmsterTime dataset improved to 57.8%. However, when faced with drastic viewpoint changes and high-frequency dynamic interference in the SF_XL dataset, due to the independence of local features, the model performance dropped significantly (from 78.4% to 57.4%), indicating that the model needs a higher-level scene understanding mechanism.

Finally, after the introduction of the global feature optimization module (module T), the system achieved a comprehensive breakthrough through the spatiotemporal semantic fusion mechanism: the model performance was significantly improved in long-term changing scenes (AmsterTime increased to 59.3%), dynamic perspective scenes (SF_XL increased to 81.1%), and changing weather conditions (at night increased to 90%, strong light interference increased to 92.9%). This hierarchical progressive optimization verifies the synergistic advantages of the “global scene modeling-local detail enhancement” dual-path mechanism, especially the key role of the Transformer architecture in establishing cross-perspective spatiotemporal associations (Nordland increased by 6.4% to 79.6%) and resisting dynamic interference (SF_XL recovered to 81.1%).

The results show the universal optimization capability of the DD–DPFE in complex VPR scenarios. The hierarchical coordination mechanism of local-global features achieves a robust representation of cross-modal scenarios through the organic integration of spatial constraints (local modules suppress feature redundancy) and temporal associations (global modules establish scene schemas). The model systematically solves the core problems of weather changes, dynamic interference response, and long-term robustness in complex VPR tasks.

#### 3.4.2. Impact of the Backbone Network on Model Performance

This subsection explores the impact of the DD–DPFE backbone model size on the performance, focusing on comparing the model performance of two backbone networks based on DINOv2&ViT-L/14 and DINOv2&ViT-B/14. The results are shown in Table 7.

Experimental data shows that DINOv2&ViT-L/14 significantly surpasses ViT-B/14 (85.16%) with an average performance of 86.89% and an absolute advantage of 1.73%. The model performs particularly well in complex dynamic scenes. Its robustness is significantly enhanced in extreme weather conditions such as SVOX-Night (+6.5%) and SVOX-Sun (+3.4%), and it achieves a 19.9% performance jump in the Nordland dataset, demonstrating its strong modeling capabilities for seasonal changes and fine-grained differences. Although ViT-B/14 surpasses ViT-L/14 (59.3%) with 80.8% in the AmsterTime dataset, suggesting the potential advantages of lightweight models under specific data distributions, ViT-L/14 leads in nine of the ten datasets, especially in scenarios with high environmental robustness requirements. Therefore, DINOv2&ViT-L/14 was selected as the default backbone network.

#### 3.4.3. Impact of Key Parameter Configurations on Model Performance

This section designs an ablation experiment to explore the mechanism of the influence of the number of Transformer stacking layers on the model’s performance in the local dynamic hierarchical fusion network and analyzes the impact of different numbers of Transformer stacking layers on the model’s performance.

**Figure 5:**
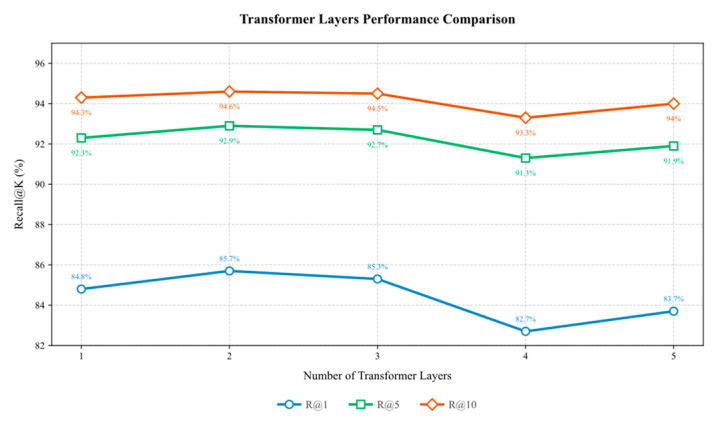
Transformer stacking layer ablation experiment.

The other parts of the model remain unchanged. Trained and tested on the MSLS dataset, the number of Transformer stacking layers was set to 1, 2, 3, 4, and 5. The experimental results are shown in Figure 5.

The results show that with the increase of fusion depth, the model performance shows a trend of increasing and then decreasing, in which the model performs better and more stably in R@1, R@5, and R@10 indexes when f=2 and f=3. Further observation shows that when the fusion depth is increased to f=4, the performance of the model decreases significantly, which we believe may be due to the redundancy of information and feature perturbation caused by the deep fusion layer, resulting in the dilution of the effective information and exacerbating the risk of overfitting, which in turn affects the model’s generalization ability. Although the result of f=5 is slightly recovered compared with that of f=4, the overall result is still lower than that of f=2 and f=3, and the difference is obvious.

In order to further verify the model’s generalization ability under different fusion depths, we selected f=2 and f=3 with better performance and f=4 with obvious degradation for full training and performed detailed tests on several datasets. The results are shown in Table 8 and Table 9.

Referring to the contents of the above table, f=2 has the best or near-optimal performance overall. On most data sets, f=2 performs better than f=3 and f=4. In the complex scene SVOX dataset, f=2 achieved the best R@1 performance under multiple weather conditions. f=3 performs slightly better in some scenes. For example, on the Nordland dataset, the R@1 of f=3 is 82.5%, higher than the 79.6% of f=2, indicating that moderate fusion helps to extract more robust features. The overall performance of f=4 declines. As the fusion depth increases, the model introduces more redundant features, which in turn affects the discriminability. For example, in the night scene, the R@1 of f=4 drops to 78.9%, which is significantly degraded compared to f=2 (90.0%). Considering the performance, stability, and generalization ability, the parameters of f=2 were selected as the default settings.

### 3.5. Visual Analytics

**Figure 6:**
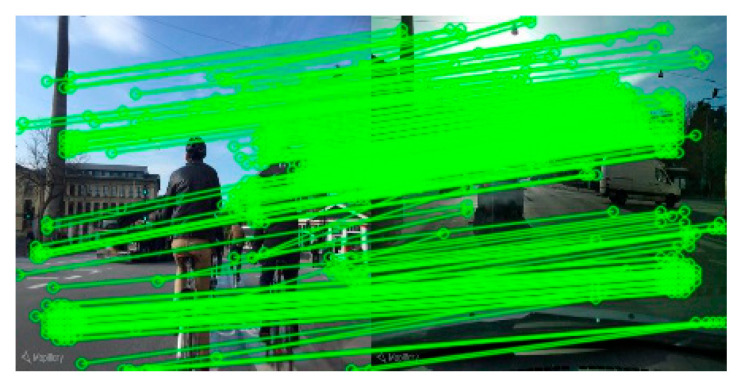
Local matching graph of the same location (271 matching points).

The local matching results of cross-view images for the same scene are shown in Figure 6. The proposed method achieves up to 271 high-confidence feature matches. This result verifies that the feature descriptors generated by the model have stronger geometric consistency and discriminability and can effectively support the refined local matching requirements in the visual position recognition (VPR) task, thereby improving the robustness of loop closure detection and pose estimation.

**Figure 7:**
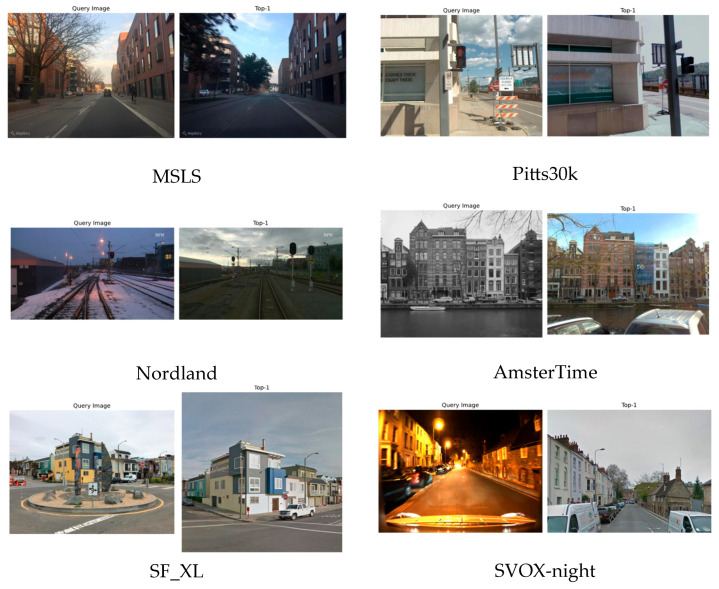

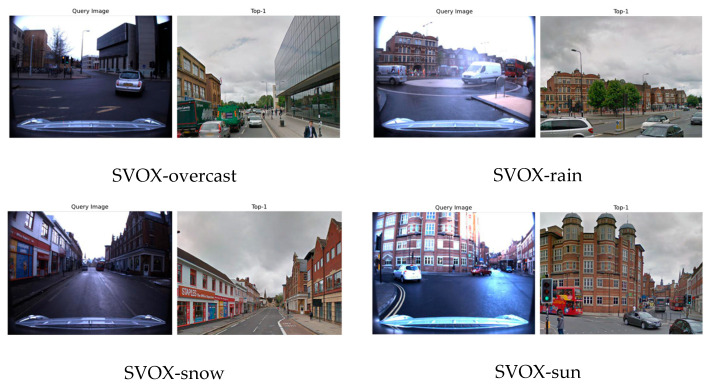
Top-1 search graph for all scenarios.

Figure 7 shows the Top-1 results retrieved by the proposed method in all scenes. As shown in the figure, all prediction results are located at the same position as the query image. For the MSLS and Pitts30k datasets, although there are weather and perspective differences between the query image and the retrieval image, the model mainly relies on the stable structure of the building for matching. In the Nordland dataset, there are significant seasonal changes (such as snow in winter and no snow in summer) and lighting differences between images; the direction of the railroad tracks and the position of street lights provide recognition bases as invariant features, but snow may block part of the track and introduce noise interference. In the AmsterTime dataset, there is a time difference between the black and white query image and the color image, but the building morphology is still the key recognition feature. The query image of the SF_XL dataset has occlusions (such as circular road signs) and large perspective changes, but the building outlines and street lights provide stable recognition information. The SVOX dataset faces challenges under different weather conditions: night scenes are insufficiently lit or partially overexposed, overcast and snow scenes have low light problems, rain scenes are blurred due to rain, and sun scenes may be overexposed due to strong light. Despite this, static elements such as building structures and intersection directions in all scenes are still the main matching basis.

## 4. Conclusions

We propose a visual place recognition method based on Dynamic Difference and Dual-Path Feature Enhancement (DD–DPFE). This method reconstructs the attention mechanism of the DINOv2 model and introduces serial and parallel adapter modules, so that the dynamic difference DINOv2 can effectively suppress the attention noise in the feature extraction process while achieving efficient migration of model parameters and task adaptation capabilities. In terms of feature enhancement, the global dynamic hierarchical fusion module is adopted to improve the cross-domain semantic modeling capability while maintaining the integrity of the initial statistical features. At the same time, the dynamic weighting mechanism of the local feature adaptive weighted aggregation module is used to effectively suppress the interference caused by repeated areas and enhance the representation accuracy of detail features. The collaborative optimization of the dual-path architecture enables the system to show significant advantages in complex environments: the R@1 accuracy rate exceeds 90% under extreme conditions such as night, rain, snow, and strong light, and achieves comprehensive leading performance on the large-scale urban Pitts30k dataset, reflecting the excellent robustness and stability in scenes with drastic changes in lighting and complex structural morphology, providing a highly reliable solution for tasks such as autonomous driving positioning and drone visual inspection.

## Figures and Tables

**Table 1:** Summary information on major evaluation data sets.

Dataset		Number		Description
		Database	Queries	
Pitts30k		10,000	6816	Illumination variation, seasonal and viewpoint change
MSLS (test)		18,871	11,084	Cross-domain difference and weather variation
Nordland		27,592	27,591	Seasonal shift and appearance change
AmsterTime		1231	1231	Long-term variation and dynamic disturbance
SF_XL (v1)		27,191	1000	Urban diversity and occlusion interference
SVOX	night	17,166	823	Low light condition and contrast degradation
	overcast	17,166	872	Lighting uniformity and low texture contrast
	rain	17,166	937	Image blur and physical interference
	snow	17,166	870	Limited texture uniqueness and scene coverage
	night	17,166	823	Low light condition and contrast degradation

**Table 2:** Details of the VPR comparison experiment.

Method	Training Dataset	Backbone Network	Dimension
CosPlace [7]	SF_XL	resnet101	512
EigenPlace [28]	SF_XL	resnet50	2048
CircaVPR [17]	Gsv_cities	DINOv2&ViT-B/14	768
R^2^former [14]	MSLS	ViT-S/12	384
SelaVPR [16]	MSLS + Pitts30k	DINOv2&ViT-L/14	1024
DD-DPFE	MSLS + Pitts30k	DINOv2&ViT-L/14	1024

**Table 3:** Comparison with existing state-of-the-art methods.

Method	Pitts30k		MSLS (Test)		Nordland		AmsterTime		SF_XL (v1)		Ave	
	R@1	R@5	R@1	R@5	R@1	R@5	R@1	R@5	R@1	R@5	R@1	R@5
CosPlace	88.2	94.7	77.8	86.0	33.2	46.2	38.9	58.1	69.3	79.8	61.5	73.0
EigenPlace	92.5	96.7	**85.7**	91.4	71.2	83.7	48.9	69.4	**83.2**	**87.4**	76.3	85.7
CircaVPR	**93.3**	96.2	83.7	91.8	**90.5**	**95.9**	**60.5**	**79.1**	75.9	83.6	**80.8**	**89.3**
R^2^former	89.2	95.9	85.5	**92.5**	60.5	66.9	28.7	43.5	68.5	72.6	66.5	74.3
SelaVPR	92.1	96.1	79.8	87.6	69.1	75.3	55.7	74.3	78.4	82.6	75.0	83.2
DD–DPFE	**93.3**	**96.9**	84.8	91.4	79.6	84.4	59.3	78.2	81.1	83.5	79.6	86.9

**Table 4:** Comparison with existing state-of-the-art methods (SVOX dataset).

Method	Night		Overcast		Rain		Snow		Sun		Ave	
	R@1	R@5	R@1	R@5	R@1	R@5	R@1	R@5	R@1	R@5	R@1	R@5
CosPlace	36.9	57.8	84.1	91.4	76	87.7	82.9	91.8	62.8	75.3	68.5	80.8
EigenPlace	59.1	77.2	93.3	97.8	89.9	96.5	93.4	97.6	86.3	94.5	84.4	92.7
CircaVPR	80	89.2	94.7	97	90.9	95.2	92.3	97.6	88.3	95.1	89.2	94.8
R^2^former	40	52	94.2	97.5	87.1	92.2	92.8	96.7	65	74.6	75.8	82.6
SelaVPR	71.6	80.7	92.5	95.8	88.3	92.2	92.8	96.1	77.6	84.1	84.6	89.8
DD–DPFE	**90.0**	**93.6**	**96.3**	**98.2**	**94.8**	**97.7**	**96.8**	**98.2**	**92.9**	**95.8**	**94.2**	**96.7**

**Table 5:** Ablation experiment (indicator is R@1).

A	W	T	Pitts30k	MSLS (Test)	Nordland	AmsterTime	SF_XL (v1)
×	×	×	92.1	79.8	69.1	55.7	78.4
√	×	×	92.1	81	73.3	49.9	76.0
√	√	×	92	80.7	73.2	50.7	57.4
√	√	√	93.3 ↑	84.8 ↑	79.6 ↑	59.3 ↑	81.1 ↑

**Table 6:** Ablation experiment (indicator is R@1, the dataset is SVOX).

A	W	T	Night	Overcast	Rain	Snow	Sun
×	×	×	71.6	92.5	88.3	92.8	77.6
√	×	×	74.2	92.1	87.1	91	80.9
√	√	×	76.7	92.8	88.8	92.5	80.9
√	√	√	90.0 ↑	96.3 ↑	94.8 ↑	96.8 ↑	92.9 ↑

Note: The corresponding “√” in the table indicates that the module is introduced into the framework, “×” indicates that this part of the module does not exist in the framework, and “↑” indicates that there is an improvement in the performance of the final result compared to the baseline model (the number of Transformer stacking layers in the global dynamic hierarchical fusion network is 2).

**Table 7:** Performance comparison of DD–DPFE under different backbone networks (indicator is R@1).

Dataset	DINOv2&ViT-L/14	DINOv2&ViT-B/14
Pitts30k	93.3	92.9
MSLS-test	84.8	83.3
Nordland	79.6	59.7
AmsterTime	59.3	80.8
SF_XL(v1)	81.1	77.9
SVOX-night	90.0	83.5
SVOX-overcast	96.3	95.9
SVOX-rain	94.8	92.7
SVOX-snow	96.8	95.4
SVOX-sun	92.9	89.5
ave	86.89	85.16

**Table 8:** Ablation comparison on benchmark datasets.

Parameter	Pitts30k		MSLS (Test)		Nordland		AmsterTime		SF_XL(v1)	
	R@1	R@5	R@1	R@5	R@1	R@5	R@1	R@5	R@1	R@5
f=2	93.3	96.9	**84.8**	**91.4**	79.6	84.4	59.3	**78.2**	81.1	83.5
f=3	**93.5**	**97.0**	83.6	90.7	**82.5**	**86.7**	59.2	76.3	80.9	83.8
f=4	92.6	96.6	81.9	89.4	69.5	74.6	**59.5**	77.3	**82.2**	**84.5**

**Table 9:** Ablation comparison on SVOX dataset.

Parameter	Night		Overcast		Rain		Snow		Sun	
	R@1	R@5	R@1	R@5	R@1	R@5	R@1	R@5	R@1	R@5
f=2	**90.0**	**93.6**	**96.3**	**98.2**	**94.8**	**97.7**	**96.8**	**98.2**	**92.9**	**95.8**
f=3	85.1	88.8	95.1	97.7	93.3	96.4	94.9	97.4	89.6	92.6
f=4	78.9	83.7	94.3	96.2	90.9	93.9	92.8	95.9	84.7	88.2

Note: Bolded values indicate optimal performance.

## Data Availability

The SF_XL dataset can be downloaded from https://github.com/gmberton/CosPlace?tab=readme-ov-file; MSLS dataset downloaded from the URL https://www.mapillary.com/dataset/places; Pitts30k dataset downloaded from the URL https://data.ciirc.cvut.cz/public/projects/2015netVLAD/Pittsburgh250k; other datasets can be downloaded from the open source project https://github.com/gmberton/VPR-datasets-downloader?tab=readme-ov-file. All datasets accessed on 6 May 2025.
